# The Implant Proteome—The Right Surgical Glue to Fix Titanium Implants In Situ

**DOI:** 10.3390/jfb13020044

**Published:** 2022-04-15

**Authors:** Marcus Jäger, Agnieszka Latosinska, Monika Herten, André Busch, Thomas Grupp, Andrea Sowislok

**Affiliations:** 1 Department of Orthopedics, Trauma & Reconstructive Surgery, St. Marien Hospital Mülheim an der Ruhr, D-45468 Mülheim, Germany; a.busch@kk-essen.de; 2 Orthopedics and Trauma Surgery, University of Duisburg-Essen, Hufelandstrasse 55, D-45147 Essen, Germany; andrea.sowislok@uni-due.de; 3 Mosaiques Diagnostics GmbH, Rotenburger Straße 20, D-30659 Hannover, Germany; latosinska@mosaiques-diagnostics.com; 4 Department of Trauma, Hand and Reconstructive Surgery, University Hospital Essen, University of Duisburg-Essen, Hufelandstrasse 55, D-45147 Essen, Germany; monika.herten@uk-essen.de; 5 Aesculap AG, Research & Development, D-78532 Tuttlingen, Germany; thomas.grupp@aesculap.de; 6 Department of Orthopedic and Trauma Surgery, Musculoskeletal University Center Munich, Ludwig Maximilians University Munich, Marchioninistraße 15, D-81377 Munich, Germany

**Keywords:** total hip arthroplasty, protein adsorption, proteomics, osseointegration, titanium, bone regeneration, biocompatibility, host-implant response

## Abstract

Titanium implants are frequently applied to the bone in orthopedic and trauma surgery. Although these biomaterials are characterized by excellent implant survivorship and clinical outcomes, there are almost no data available on the initial protein layer binding to the implant surface in situ. This study aims to investigate the composition of the initial protein layer on endoprosthetic surfaces as a key initiating step in osseointegration. In patients qualified for total hip arthroplasty, the implants are inserted into the femoral canal, fixed and subsequently explanted after 2 and 5 min. The proteins adsorbed to the surface (the implant proteome) are analyzed by liquid chromatography–tandem mass spectrometry (LC-MS/MS). A statistical analysis of the proteins’ alteration with longer incubation times reveals a slight change in their abundance according to the Vroman effect. The pathways involved in the extracellular matrix organization of bone, sterile inflammation and the beginning of an immunogenic response governed by neutrophils are significantly enriched based on the analysis of the implant proteome. Those are generally not changed with longer incubation times. In summary, proteins relevant for osseointegration are already adsorbed within 2 min in situ. A deeper understanding of the in situ protein–implant interactions in patients may contribute to optimizing implant surfaces in orthopedic and trauma surgery.

## 1. Introduction

Cementless total hip replacement of titanium (Ti) alloys is a worldwide standardized surgical procedure that improves mobility and quality of life, predominantly in the elderly population. In addition, plates, nails, screws and wires made of Ti belong to the daily standard repertoire of orthopedic and trauma surgeons. Compared to other biomaterials, Ti alloys have shown excellent clinical results in most patients [1].

In contrast to an ample number of in vitro and in vivo studies investigating Ti osseointegration, along with numerous clinical studies, the local humoral and cellular interactions governing processes in the human *situs* remain unclear. In particular, initial protein adsorption is currently the subject of research. In this context, the in situ implant proteome was described in a pilot study for the first time in 2019 [2]. As a major result, not only extracellular but also intracellular proteins were detected on the implant’s surface (Ti-6Al-4V, cpTi). Predominantly, high amounts of cell-free hemoglobin were documented. Moreover, the implant proteome did not reflect a substantial contribution from the plasma proteome, as hypothesized by other investigators [2,3]. Other studies have demonstrated that not only osteoblastic precursors but also immunocompetent cells, such as macrophages and granulocytes, migrate early to the implantation site [4,5]. However, it is unclear if proteins adhered to the implant will have chemotactic effects on immune cells or if proteins from the immune system will adhere to the implant’s surface itself. According to the Vroman effect, the most mobile proteins generally adsorb first to the biomaterial, and those are subsequently replaced with less mobile proteins with a higher surface affinity [6,7]. The adsorption of proteins to the implant’s surface in situ has not been fully explored. Thus, it is still unclear which proteins adhere to the implant’s surface and how their abundance changes over time and on different surface structures. Therefore, in this study, we aimed to characterize the protein adsorption onto the Ti implant’s surface towards answering the following two emerging questions: (1) is there a difference in the protein adsorption on the implant’s surface over time (from 2 to 5 min), and (2) if adsorption varies between differently structured Ti surfaces (rough and smooth). Our data provide a deeper insight into the understanding of local bone/bone-marrow-implant interactions in the human situs, allow a better description of the osseointegration process at the molecular level, and may lead to further improvement and design of orthopedic implants in the future.

## 2. Materials and Methods

### 2.1. Implants

A commercial Ti-6Al-4V femoral stem (BiCONTACT™ stem, Braun Aesculap, Tuttlingen, Germany) was used. This meta- and diaphyseal press-fit implant has been successfully applied for more than 20 years and has shown excellent clinical long-term results [8,9]. The implant contains two different surface structures [10]. Briefly, the proximal part is rough as it is covered with a 0.35 mm thick plasma-sprayed layer of commercial pure Ti (cpTi) (Plasmapore™, porosity 35%, pore size 50–200 µm), while the distal part of the implant is smooth as it was treated with glass bead blasting.

### 2.2. Probands, Surgical Technique and Implant Retrieval

Probands: Twelve adult patients in advanced stages of osteoarthritis scheduled for total hip replacement were included. All patients had given their informed consent. Exclusion criteria were septic conditions, active neoplasm or other consuming diseases (autoimmune diseases) or coagulopathy. The study has been approved by the Ethical Commission of the University Duisburg-Essen (AZ 17-7844-BO).

Surgical technique and implant retrieval: An antero-lateral approach to the right hip joint was performed (Harding–Bauer approach) [11,12] by one surgeon (M.J.). Following femoral neck resection, the femoral canal was prepared by two different rasps following the manufacturer’s manual (A-/B-Osteoprofiler). Here, instead of removing the spongy bone, local bone was preserved and compressed by controlled mallet stokes, allowing a maximum of stability. Afterwards, an original BiCONTACT™ stem was implanted and controlled by fluoroscopy image intensifier. After an in situ time of either 2 or 5 min the stems were explanted via no touch technique. The stems were washed twice for 1 min with saline, packed in a sterile plastic bag and quick-frozen in liquid nitrogen for transport and subsequent storage at −80 °C. In total, 13 implants were analyzed.

Removal and Collection of the Adsorbed Protein Layer from Implants: Protein removal from both implant surfaces (rough and smooth) followed a standard protocol using the following three different elution buffer solutions at room temperature: (i) buffer A (4% SDS, 1 M DTT, 0.1 M Tris-HCl pH 7.6), (ii) buffer B (4% SDS, 1 M DTT, 1 M NaCl, 0.1 M Tris-HCl pH 7.6) and (iii) buffer C (4% SDS, 0.1 M DTT, 1 M NaCl, 0.1 M Tris-HCl pH 7.6), as described in detail in [2].

### 2.3. Protein Quantification

Before protein quantification, the eluted protein samples of the hip implant were concentrated by TCA precipitation, as described previously [2]. For eluates of the smooth surface, the protein content was concentrated three-fold, while the eluates of the rough surface remained unconcentrated. Protein quantification was performed using the Lowry method [13] for eluates of the rough surface as described before [2]. For eluates of the smooth surface, a modified micro BCA assay [14] (Thermo Fischer Scientific, Rockford, IL, USA) was used. In brief, 50 µL of the protein sample was mixed with 250 µL of the BCA reagent (4.8 mL solution B + 200 µL solution C + 5 mL solution A) and incubated at 60 °C for 1 h. Afterward, the samples were measured at 570 nm in a plate reader (Multiskan Ascent, Thermo-Fischer, Rockford, IL, USA). The protein concentrations were measured in triplicates at three different dilutions (1:2, 1:3, 1:4).

### 2.4. Proteome Analysis

In total, 1 mL aliquots of the lyophilized samples were re-suspended in 10 mM dithioerythritol (DTE) solution. After initial SDS-PAGE analysis, samples from the smooth surface were further concentrated using Amicon filters (10 kDa cut-off) so that a similar number of proteins could be loaded onto the second SDS-PAGE. Here, 20 µL of each sample was loaded onto the gel and prepared according to the GeLC-MS method, as described in [15]. LC-MS/MS analysis was performed as described previously [2,16]. Briefly, liquid chromatography was performed with 5 µL protein digest loaded onto a 0.1 × 20 mm 5 μm C18 nano trap column (Dionex Ultimate 3000 RSLS nanoflow) and subsequently separated on an Acclaim PepMap C18 nano column 75 μm × 50 cm (Dionex, Sunnyvale, CA, USA) using an 8 h gradient. The LC was connected to an Orbitrap Velos FTMS (Thermo Finnigan, San Jose, CA, USA) via a Proxeon nanospray.

Evaluation of proteomics data: Protein identification was performed with Proteome Discoverer 1.4 (Thermo Scientific) using the SEQUEST search engine, as described previously [2]. In brief, a protein search was performed against the Swiss-Prot human protein database [17]. The following search parameters were applied: (i) precursor mass tolerance: 10 ppm and fragment mass tolerance: 0.05 Da; (ii) full tryptic digestion; (iii) maximum missed cleavage sites: 2; (iv) static modifications: carbamidomethylation of cysteine; (v) dynamic modifications: oxidation of methionine and proline. After completing the analysis of individual RAW MS files, proteomics data were exported at the peptide level using the following filters: (i) peptide rank up to 5; (ii) mass deviation (ΔM) ± 5 ppm; (iii) peptide grouping was enabled, and protein grouping was disabled. Data were further evaluated using the clustering approach, together with previously acquired RAW data [2], as described before with some modifications [2]. Briefly, after the calibration of the retention time against the one sample selected as reference, peptides from different proteomics runs were grouped (“clustered”) based on the predefined window of mass (±5 ppm) and retention time (±5% of the peptides’ retention time). Each cluster contains a group of peptides for which the sequence (belonging to the cluster) with the highest sum of Xcorr values was reported as reference. When the same Xcorr sum was reported, the frequency of sequence was considered as a second criterion. The sequences were selected among those with Xcorr above 1.9 and without proline hydroxylation on the non-collagen proteins. Quantification of proteomics data was based on the peak area that uses the precursor ions for estimation of relative abundance. Individual peptide peak areas were normalized using the following part per million (ppm) normalization: normalized peak area = (peptide peak area/total peak area in a sample) × 1,000,000. Protein abundance in each sample was calculated as the sum of all normalized peptide areas for a given protein, as described previously [2].

Statistical analysis: Statistical analysis of the proteomics data was performed using the non-parametric Wilcoxon test, which was shown to be more appropriate for the proteomics data [18]. In addition, data normality was assessed using Kolmogorov–Smirnov test, supporting that in most cases data are not normally distributed. Paired statistics were performed only when comparing proteins between the rough and smooth surfaces at different exposure times. The following comparisons were made: (1) 2 min and 5 min exposure at the rough surface, (2) 2 min and 5 min exposure at the smooth surface, (3) smooth and rough surface at 2 min exposure and (4) smooth and rough surface at 5 min exposure. Differentially abundant proteins were defined separately in each comparison based on the following criteria: proteins identified in at least 60% of samples in at least one group, for which nominal significant difference in protein abundance between conditions was observed (unadjusted *p* < 0.05). Linear regression was applied to assess the relationship between protein abundance across different conditions.

### 2.5. Annotation of Proteomics Data and Bioinformatics Analysis

Identified proteins were extensively annotated using different resources and previously published data. Specifically, for information on the plasma proteome, data were retrieved from Plasma Proteome Database [19] and literature. The latter included a list of 945 proteins (≥10 peptide spectrum matches and ≥ 2 peptides) described by Farrah et al. [20], 1175 proteins described by Anderson et al. [21] and 713 protein groups (≥2 peptides) identified by the report from Geyer et al. [22]. The implant proteome was also compared with the red blood cell proteome comprised of 2309 protein groups (≥2 peptides) [23]. The Human Protein Atlas [24] served as a source for information on the subcellular locations (including also secretome locations), while information about human extracellular matrix (ECM) proteins was retrieved from MatrisomeDB [25,26]. Information regarding protein length, mass and function was retrieved from the Uniprot database [17].

Pathway enrichment analysis was conducted using the online tool ShinyGO v0.66 [27,28] with a curated Reactome database used as an ontology source. Significantly enriched pathways were defined based on the *p*-value cut-off (false discovery rate (FDR)) below 0.05.

## 3. Results

### 3.1. Patients Characteristic

**Figure 1:**
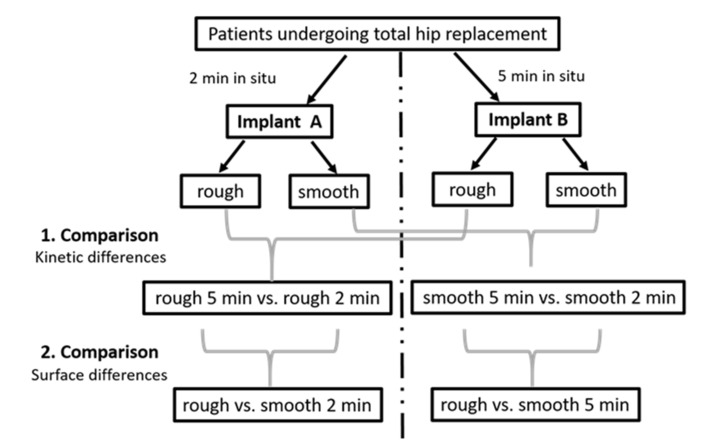
Visualization of our study design with clarification of pairwise comparisons.

Implants from 12 patients, mainly females, with advanced osteoarthritis, with an average age of 73.5 were investigated. An overview of the demographic and clinical data of the study cohort is displayed in Table 1. Among these 12 patients, 1 patient provided 2 implants, and each of the other 11 patients provided 1 implant. Thus, 13 implants in total were included in the study and led to the generation of 26 implant eluates (13 from rough and 13 from smooth implant surfaces), for which proteomics analysis was conducted. Twenty-six proteomics datasets covered 20 newly acquired and 6 previously generated datasets [2]. A graphical representation of the study design is presented in Figure 1.

The time of surgery was comparable in all patients. The pre- and post-operative serum and blood parameters were uneventful and comparable (Table 2).

### 3.2. Characterization of the In Situ Implant Proteome

Proteomic profiles of 26 implant eluates were analyzed, including 14 (7 per surface) obtained after 2 min and 12 eluates (6 per surface) obtained after 5 min in situ. To investigate the proteomic changes between different exposure times and surfaces, the proteomic profiles were separated into the following four groups (conditions): (1) rough surface at 2 min exposure (*n* = 7), (2) smooth surface at 2 min exposure (*n* = 7), (3) rough surface at 5 min exposure (*n* = 6) and (4) smooth surface at 5 min exposure (*n* = 6). An overview of the analyzed samples is provided in Table 3.

**Figure 2:**
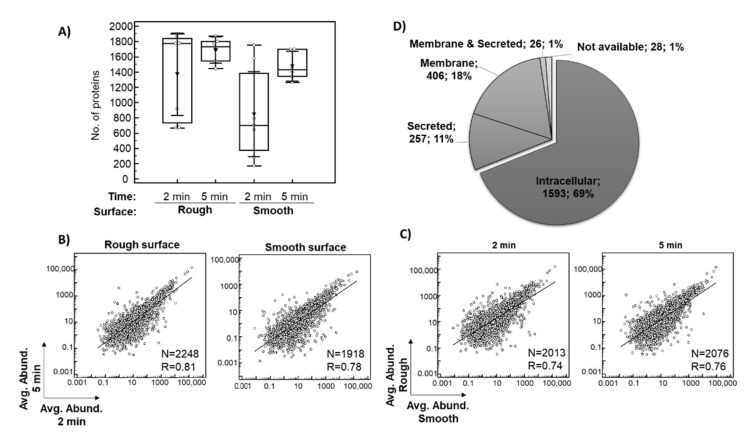
Overview of the implant proteomics data. The box plot shows the distribution of the number of proteins identified (≥2 peptides) per sample across the analyzed conditions, with an average value indicated with triangle (**A**). Scatter diagrams with regression lines showing the relationship between averages of the relative abundance of each of the identified proteins for eluates collected after 2 and 5 min exposure in situ (**B**), as well as eluates from smooth and rough surfaces are presented (**C**). A pie chart shows the subcellular localization of the identified proteins based on the data provided in Human Protein Atlas (**D**). Abbreviations: Avg. Abund.–ppm normalized protein abundance, R–coefficient of correlations, N–number of proteins included in the analysis (common proteins between the investigated conditions).

Only proteins identified based on at least two peptides in the entire dataset of 26 eluates were considered for subsequent investigation. In total, 1367 proteins after 2 min and 1687 proteins after 5 min of exposure in situ were identified on average per eluate from the rough surface. The average number of proteins identified per eluate from the smooth surface was 847 after 2 min and 1476 after 5 min in situ (Figure 2A). In general, more proteins were identified after a 5-min exposure as well as on the rough surface, which is consistent with the protein concentrations of the sample (Table 3). There was a strong relationship between the averages of the relative abundance of each of the identified proteins after 2 and 5 min in situ (Figure 2B, *p* < 0.0001) and between the smooth and rough surfaces (Figure 2C, *p* < 0.0001), indicating a good consistency of the results.

The proteomic analysis resulted in the identification of a total of 2310 proteins based on at least two peptides (Supplementary Table S1). Among these 2310 proteins (Figure 2D), 69% (1593) were of intra-cellular origin, 18% (406) were assigned to the membrane fraction, while 11% (257) were classified as secreted, with around 50% being secreted into the blood. Moreover, 8% (183) of the implant proteomes belonged to the group of ECM proteins (core ECM or ECM-associated proteins). Depending on the plasma reference set investigated, between 4% and 18% of implant proteins were annotated as plasma proteins, while 25% of all proteins belonged to the red blood cell proteome (Supplementary Table S1). A similar distribution of proteins was observed for each analyzed condition. The 10 most abundant proteins within this classification are listed in Supplementary File A and are among the 200 proteins with the highest peptide counts. Table 4 lists the top 10 most abundant proteins in the pooled analysis of 26 samples. Highly abundant intracellular/membrane proteins, e.g., include different hemoglobin subunits, actin cytoplasmic 1, myosin-7, carbonic anhydrases, spectrin beta chain and peroxiredoxin 1. Proteins secreted into the blood included serum albumin, alpha-1-antichymotrypsin, fibrinogen chains, alpha-1-antitrypsin, serotransferrin, alpha-2-macroglobulin, apolipoproteins and complement C3, while ECM proteins were represented by different types of collagen.

The pathway enrichment analysis of the above-mentioned top 200 proteins was performed to place proteomic findings in their biological context and indicate pathways represented by the proteins that adsorb to the implant surface. The 25 most significantly enriched pathways were related to ECM organization (e.g., pathways involved in collagen formation, ECM proteoglycans, integrin cell surface and non-integrin membrane-ECM interactions), and hemostasis (platelet activation, signaling and aggregation). In addition, the pathways related to signal transduction (signaling by receptor tyrosine kinases), metabolism of proteins and the immune system (neutrophil degranulation) were identified as listed in Table 5.

### 3.3. Changes in Protein Adsorption on Titanium Implants

#### 3.3.1. Proteomic Differences between 2 and 5 Min In Situ

**Figure 3:**
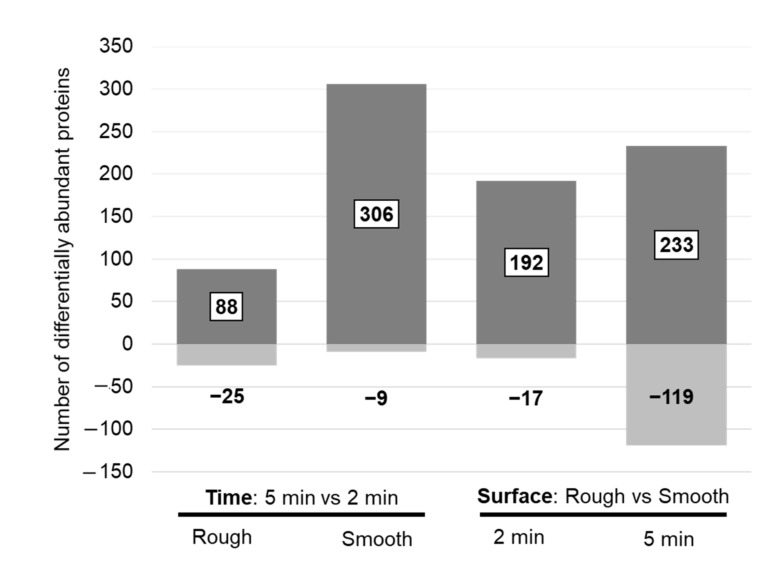
Number of differentially abundant proteins when comparing protein changes between two exposure times on a defined surface (**left panel**) and between surface properties at a defined time point (**right panel**). Discrimination for proteins with an increased (dark grey = up) and decreased (light grey = down) abundance in the “case condition” per comparison is given.

Pairwise statistical comparisons revealed 113 significantly different proteins when comparing eluates from the rough implant side after 2 and 5 min in situ, whereas 315 proteins were significantly different on the smooth surface. For most of these proteins, the abundance increased after 5 min (Figure 3, left panel). Among the 200 proteins with the highest peptide number, only a few proteins on the rough surface were significantly changed, whereas the most striking changes were observed on the smooth surface (Table 6, Supplementary Table S1). The proteins with increased abundance after 5 min in situ on the smooth surface included some of the intracellular and ECM proteins with high abundance (Table 4), including hemoglobin subunit beta, collagen alpha-1(XXII) chain and collagen alpha-1(III) chain. Many of these differentially abundant proteins also showed statistical significance when comparing surface properties (Table 6).

The pathway enrichment analysis of all significantly different proteins (5 vs. 2 min in situ) revealed seven enriched pathways on the smooth surface. The proteins belonging to these significantly enriched pathways were mainly related to ECM organization, hemostasis and NCAM signaling (Table 7). For the rough surface, no significantly enriched pathways were found.

#### 3.3.2. Proteomic Differences between the Smooth and the Rough Surfaces

Pairwise statistic comparisons (rough vs. smooth) revealed 209 differentially abundant proteins after 2 min and 352 proteins after 5 min in situ. In general, protein abundance was higher on the rough surface (Figure 2). Several of these proteins, belonging to the top 200 proteins with the highest peptide number, were among the top 10 highly abundant intracellular/membrane, secreted and ECM proteins (Table 8). After 2 min in situ, the proteins with increased abundance on the rough surface were the following: hemoglobin subunit delta, myosin-7 (intracellular/membrane), alpha-1-antitrypsin, alpha-2-macroglobulin, complement C3, apolipoprotein A-I (secreted to blood), collagen alpha-1(II) chain, collagen alpha-1(XXII) chain, collagen alpha-6(IV) chain and collagen alpha-1(III) chain (ECM proteins). After 5 min in situ, hemoglobin subunits (beta, alpha, delta), actin, carbonic anhydrase 1 and 2, myosin-7, peroxiredoxin-2, vimentin (intracellular/membrane), alpha-1-antitrypsin, fibrinogen gamma chain, apolipoprotein B-100 and A-I, haptoglobin (secreted to blood) were increased on the rough surface. Furthermore, 16 of these differentially abundant proteins (Table 8) experienced a significant change in abundance after 2 min and 5 min in situ, with most showing increased abundance on the rough surface. These proteins included tropomyosin beta chain, myeloperoxidase, malate dehydrogenase, hemoglobin subunit delta, histone H4 and myosin-7 (belonging to intracellular proteins), complement factor B, alpha-1-antitrypsin, apolipoprotein A-I, angiotensinogen, complement C4-B (proteins secreted into the blood), collagen alpha-1(XV) chain, collagen alpha-1(XIII) chain and decorin (ECM proteins).

The pathway enrichment analysis of all significantly different proteins (rough vs. smooth) revealed multiple enriched pathways for both time points (Table 9). After a shorter incubation time of 2 min, significantly enriched pathways mainly related to extracellular matrix organization (mainly involving collagens), the immune system (neutrophil degranulation, signaling by interleukins, complement cascade), hemostasis (platelet activation) and muscle contraction. After a prolonged incubation time of 5 min, the most significantly enriched pathways were related to metabolism (carbohydrate metabolism), the immune system (neutrophil degranulation, signaling by interleukins), hemostasis (platelet activation) and signaling (by MAPK family cascade).

## 4. Discussion

The local humoral and cellular mechanisms in situ after the first intraoperative bone contact of an implant until load-stable osseointegration are complex and have not yet been fully elucidated in detail. They follow a strict sequence of protein adsorption, cellular migration, proliferation, biomineralization and subsequent bone remodeling processes [29,30]. In our previously published study, we were able to disprove the generally accepted hypothesis of immediate adsorption of plasma proteins to the implant surface in situ [2]. We reproduced these data with a significant correlation between the average protein abundance (*p* < 0.0001). However, in this study, we were interested in the kinetic changes of the initial protein layer and its function in osseointegration. According to the Vroman’s effect, in which smaller proteins with higher concentrations adsorb to the surface first and are later replaced by larger proteins with higher binding affinities [31], we expected differences in the protein composition of eluates from a specific surface with increasing incubation time. Investigating the proteome of the implant eluates at two different time points revealed that both surfaces assimilated their protein portfolio over time, with the protein abundance being higher on the rough implant surface (Figure 2). To better understand the molecular mechanisms underlying osseointegration, proteomic results were placed in the context of biology, discovering pathways belonging to ECM organization, hemostasis, signal transduction, metabolism of proteins and the immune system. Most of these pathways were also detected when analyzing changes between smooth and rough surfaces, while only a few pathways (mainly those involved in ECM organization) were significantly enriched on the smooth surface when comparing the 2 and 5 min exposures in situ (Supplementary Table S2). These findings suggest a higher relevance of surface structure to the osseointegration process rather than that of time. The evaluation of the findings in the biological context is discussed in detail in the following sections.

### 4.1. Proteins of the Bone ECM and Their Distribution within the Implant Proteome

ECM is a basic component of tissues and organs and provides both structural and non-structural support that leads to osseointegration. This is supported by our findings showing a significant enrichment in the pathways involved in ECM organization, represented by collagenous and non-collagenous proteins. Since collagen accounts for 90% of the organic ECM of bone, collagen biosynthesis and collagen biochemistry may be key determinants of osseointegration [32]. Among bone ECM proteins, collagen type I, typically organized into collagen fibrils consisting of triple helices of polypeptides, is the most abundant. The collagen fibrils interact with other collagenous and non-collagenous proteins to assemble higher-order fibril bundles and fibers. The fiber diameter and fibrillogenesis are regulated by collagen type III and V, which are present in smaller amounts in the bone ECM [33]. Within the implant proteome, various collagen types were found to adsorb to the implant surface, with the abundance significantly affected by either incubation time (Table 6) or surface properties (Table 8). While the abundance of collagen type I was similar on both surfaces after a 2 min exposure in situ and did not change substantially with longer incubation time, the abundance of collagen type III was increased on the rough surface, followed by further enrichment on the smooth surface with longer incubation time (5 min). Collagen type V also accumulated on the smooth surface during prolonged exposure (Table 6). The non-collagenous proteins of bone ECM constitute a large group of diverse proteins that are non-stoichiometric with type I collagen but of great importance for bone physiology. Mutations in a number of these proteins result in bone abnormalities [34]. Within the implant proteome, some of the most common members of these proteins have been identified as having a higher sensitivity to surface properties than to incubation times. In the group of small leucine-rich proteoglycans, which are extracellular secreted proteins involved in all phases of bone formation, including cell proliferation, osteogenesis, mineral deposition, and bone remodeling [33], biglycan, asporin, decorin and lumican were found, with the latter two proteins being among the 200 most frequently identified proteins (Table 8). While biglycan showed strong binding to both surfaces, which was not significantly affected by the exposure time, the abundance of both asporin and lumican was increased on the rough surface compared to the smooth surface after prolonged incubation in situ. Along these lines, the decorin level was increased on the rough surface in comparison to the smooth surface, and its abundance was not affected by incubation time. Glycoproteins such as thrombospondins, fibronectin and vitronectin were also part of the implant proteome, with fibronectin belonging to the top 200 proteins identified with the highest number of peptides (Table 8). Thrombospondins are expressed by osteoblasts and are present at different stages of bone maturation and development [34]. The abundance of thrombospondin 1 and 2 was stable on both implant surfaces and did not increase with time, while thrombospondin 4 showed an increase after 5 min on the rough surface in comparison to the smooth surface. Fibronectin and vitronectin are plasma proteins that interact with other ECM proteins and are crucial for collagen-matrix assembly [35]. In the implant proteome, fibronectin that is produced during the early stages of bone formation and upregulated in osteoblasts [34] is increased after 2 min of incubation on the rough surface, but it does not significantly increase with time (Table 6). Whereas after a 5 min exposure in situ, the abundance of vitronectin, which is found at low levels in a mineralized matrix [34], is significantly reduced on the rough in comparison to the smooth surface. The enzymes involved in the posttranslational modification of collagen, or its enzymatic degradation may be of further importance when considering the organization of bone ECM. The formation of inter- and intra-molecular crosslinks of collagen is regulated by posttranslational hydroxylation and oxidation of specific lysine residues catalyzed by lysyl-hydroxylases and oxidases [32]. Some of these proteins, such as prolyl 4-hydroxylase subunit alpha-, lysyl-oxidase 1 and 2, were among the proteins adsorbing to the implant surface. Their abundance (although low) was stable and not affected by incubation time or surfaces. Another important class of proteins concerning the degradation of extracellular matrix components are the zinc-dependent matrix metalloproteases (MMPs) that are also involved in bone resorption by osteoclasts [36]. In our dataset, MMP21 does not show a significant increase over time, and its abundance was stable independent of the surface. MMP9 is increased on the rough surface, albeit only at the 2 min time point.

### 4.2. Hemostasis and Inflammation

Hemostasis and inflammation are interconnected physiological processes, with inflammation leading to hemostasis activation that, in turn, influences the inflammation. A pathway enrichment analysis revealed that the top 200 proteins that adsorb to the implant’s surface (Table 5) are involved in hemostasis, the complement cascade and neutrophil degranulation. Those pathways were also found to be significantly enriched based on the analysis of differentially abundant proteins between the two implant surfaces (Table 9) but were not significantly different when comparing the two incubation times (Table 7). As defined by Alberktsson et al., osseointegration is a mild inflammatory response leading to an integrated implant with a bone-implant interface that remains in a state of equilibrium, susceptible to changes in the environment [37]. Disturbance in this foreign body equilibrium may lead to peri-implant bone loss through reactivation of inflammation, the formation of foreign body giant cells and the activation of osteoclastogenesis [38].

#### 4.2.1. Proteins Potentially Involved in Hemostasis and Neutrophil Activity

Enhanced hemostasis is a natural reaction to prevent excessive blood loss and maintain blood flow to the rest of the body as an answer to the damage of blood vessels in the periosteum, endosteum and surrounding soft tissues during surgery [39]. Activated platelets are the first cells to respond to wound healing through the processes of adherence, aggregation and degranulation [40]. In vivo, the disruption of the continuity of the endothelial layer and the exposure of the underlying subendothelial matrix leads to the activation of platelets through the interactions between collagen, fibronectin, von Willebrand factor and various glycoprotein receptors on the platelets [41]. Interestingly, most of these proteins were of higher abundance on the rough surface after a 2 min exposure in situ, with collagen and fibronectin being among the top abundant 200 proteins (Table 8). The abundance of von Willebrand factor was reduced on the rough surface in comparison to the smooth surface after 2 and 5 min in situ, and its abundance was further increased on the rough surface with longer incubation (5 min). Besides their role in hemostasis, platelets were also found to be involved in the activation of the immune system through their surface receptors and their granules, which contain a plethora of biologically active products [42]. The physical interactions between neutrophils and platelets are triggered by the expression of p-selectin on activated platelets that can bind to p-selectin glycoprotein ligand-1 on the surface of neutrophils. Additionally, the secretion of CD40 ligand by platelets has been shown to upregulate integrin expression on neutrophils, and the secretion of serotonin and platelet factor 4 leads to neutrophil recruitment and adhesion [41,42]. We could not detect adhesion of p-selectin to the Ti implant but found platelet factor 4 adsorbed to the implant surface in situ, albeit at a low abundance with no significant changes concerning surface properties and exposure times.

As aforementioned, the pathway analysis also suggested the activation of neutrophils and their degranulation. Along these lines, the leucocyte concentration was found to increase after surgery in the blood levels of all patients (Table 2). Neutrophils, together with basophils and eosinophils, belong to polymorphonuclear leukocytes, constitute 40–65% of the white blood cell population and are hallmarks of acute inflammation [43,44]. Immediately following tissue injury, these cells are recruited and exert anti-microbial activity via degranulation, enzymatic release, phagocytosis of foreign substances and debris and the production of large DNA-based fiber networks called neutrophil extracellular traps [5]. Upon activation, neutrophils release toxic mediators including elastase, myeloperoxidase, cathepsins and defensins from their primary granules [45]. These proteins were also present in the implant proteome, with myeloperoxidase and elastase among the most striking differences when comparing surfaces (Table 8). Myeloperoxidase and cathepsin G were found significantly enriched on the rough surface (both after 2 and 5 min). Myeloperoxidase catalyzes the formation of reactive oxygen species, such as hypochlorous acid (HOCl) [46], while cathepsin G stimulates the production of cytokines and chemokines responsible for the activation and mobilization of immune cells to the site of pathogen or tissue damage [47].

#### 4.2.2. Possible Neutrophil Recruitment through DAMPs and the Complement System

The proteomic analysis also indicated another possible mechanism for neutrophil recruitment. Because of surgery-induced tissue damage, necrotic cells release self-derived molecules that are either altered or relocated from their normal cellular compartment [48]. Those damage-associated molecular patterns (DAMPs) are known to activate neutrophils and trigger a sterile inflammatory response [49]. The DAMPs include extracellular ATP, formylated peptides and DNA of mitochondrial origin, nucleic acids, heat shock proteins, S100 proteins, high mobility group protein B1 and altered extracellular matrix components, such as hyaluronan [48,49]. The members of these protein families were also found in the implant proteome. Hyaluronan, high mobility group protein B1, numerous heat shock proteins and S100A8 and S100A11 were identified on the implant surface, with their abundance on the implant surface not being affected by the surface type or time. However, the 10 kDa heat shock protein was found to be significantly increased on the rough surface after 5 min in situ, and its abundance was also accumulated on the smooth surface with a longer incubation time. Heat shock protein HSP 90-beta was only increased with a longer incubation time on the smooth surface. In addition, the protein S100A9, known to induce neutrophil chemotaxis and adhesion [50], was found to be consistently elevated on the rough in comparison to the smooth surface after both 2 min and 5 min of exposure in situ.

Another immunologic recruitment mechanism for neutrophils is the activation of the complement system. Biomaterials, including Ti implants, are known to activate the complement cascade through the classical pathway, with further amplification through the alternative pathway [51]. Several complement factors are also detected in the implant proteome. Among them, complement factor B (CFB), C3, C4 and C5 were found significantly enriched on the rough and smooth surfaces after an exposure time of 2 min in situ (Table 8). In addition, the abundance of CFB and C4 was also increased on the rough surface after 5 min exposure, while C5 showed an increase in abundance on the smooth surface after 5 min in comparison to 2 min. Furthermore, the abundance of complement C1q subcomponent subunit B was also significantly increased with prolonged incubation on the smooth surface, while C8 showed an increase in the abundance on the rough surface after 5 min in situ. Interestingly, the CFB and C4 proteins are involved in the classical (C4) and alternative (CFB) complement activation pathways. However, the question of whether the complement system is activated cannot be answered with confidence based on the proteomics data.

Moreover, bone cells can produce complement factors and are in turn targets of activated complement [51]. Osteoblasts can produce C3 and C5, while osteoclasts can release activated C5a. The anaphylatoxins C3a and C5a are not only chemotactic for neutrophils but also for osteoblasts and their mesenchymal stem cell precursors [52,53]. In osteoblasts, they stimulate the release of inflammatory cytokines, including IL-6 and IL-8. Additionally, C5a can stimulate the secretion of osteoclastogenic factors [51,52]. This suggests that the activation of the complement system might also directly influence bone healing through its interaction with bone cells. Enhancing osteoclastogenesis might increase bone resorption while recruitment of osteoblasts and their precursor cells might favor bone formation [52].

### 4.3. Limitations of Our Study

The sample size for each group was low and did not allow adjustment for multiple testing. Similarly, an evaluation of differences in the protein adsorption between male and female patients could not be performed. Since females are more likely to have bone-related diseases (e.g., osteoporosis and osteoarthritis), the influence of gender on protein adsorption at the implant surface will be investigated in a follow-up study. Furthermore, we are aware that incorrect sequence assignment may occur on occasion, especially in the case of low abundant peptides where the spectral quality is lower. However, the risk of false protein identification is reduced by excluding proteins identified based on one peptide only. For the assignment of proteins to the plasma proteome, we used the Plasma Proteome Database downloaded in June 2018, for which the download option in the online database has been temporarily unavailable, so newer additions to the database were not considered. Although we examined the implant proteome at two different time points, our study documents only a snapshot of protein adsorption after a short period. However, longer incubation times in situ during implant surgery are not possible for ethical reasons. Since some animal studies indicate that low protein intake might affect the osseointegration of Ti implants [54], the dietary habits of our patients, which were not considered, might have impacted the results of our study.

## 5. Conclusions

Our study indicates that the proteins adsorbed to orthopedic implants may allow insights into the molecular mechanisms directly taking place in humans after biomaterial insertion. We were able to match the proteins identified in the eluates from the implant’s surface to pathways implicated in osseointegration. Despite the short exposure time of 2 to 5 min in situ, we were able to comprehend remodeling of the bone extracellular matrix and the onset of an inflammatory response as key steps of osseointegration. The proteins adsorbed to the implant’s surface may show a chemotactic effect on immune cells and other immune cell-derived proteins adhered to the implant’s surface. The composition of the adsorbed proteins indicates that bone healing immediately begins at a molecular level after implantation.

## Figures and Tables

**Table 1:** Baseline demographic and clinical data. Continuous data are shown as mean and standard deviation and categorical data as number and percentage (%).

Variable		Value
Number of patients		12
Age (years)		73.5 ± 7.34
Male		3 (25%)
BMI		27.6 ± 3.01
Surgery site	left	5 (42%)
	right	7 (58%)
Surgery time (min)		88.5 ± 7.56
Hospital time (days)		11.4 ± 3.18
**Comorbidities**		
Hypertension		6 (50%)
ACVB (aorto-coronary-vein-bypass)		2 (16.6%)
Hypothyreosis		2 (16.6%)
Rheumatoid arthritis		2 (16.6%)
Adipositas		1 (8.3%)
Diabetes mellitus		1 (8.3%)

**Table 2:** Patients’ pre- and post-operative serum parameters (*n* = 12). These parameters were assessed one day before surgery (−1), on the day of surgery (0) and two days after surgery (2). Abbreviation: pTT–partial thromboplastin time.

Variable	Time Point	Mean	Standard Deviation	Normal Values
**Sodium (mmol/L)**	−1	139.8	4.2	
	0	138.9	3.7	136–145
	2	138.4	3.7	
**Potassium (mmol/L)**	−1	4.6	0.3	
	0	4.2	0.3	3.5–5.1
	2	4.0	0.3	
**Leucocytes (1/nL)**	−1	7.2	1.7	
	0	11.8	2.8	4.0–11.0
	2	9.6	2.9	
**Hemoglobin (g/dL)**	−1	14.0	1.2	
	0	11.8	1.9	11.6–16.3
	2	10.2	1.5	
**Thrombocytes (1/nL)**	−1	255.8	63.3	
	0	213.7	38.2	140–320
	2	195.2	40.5	
**Orotein (g/L)**	−1	67.1	5.9	
	0	58.3	8.1	64–83
	2	54.0	3.4	
**Quick (%)**	−1	101.4	23.9	
	0	91.0	12.9	70–130
	2	96.5	16.8	
**pTT (s)**	−1	26.6	3.8	
	0	26.1	2.4	24–32.2
	−1	28.2	1.9	
**Fibrinogen (mg/dL)**	−1	324.3	66.2	
	0	312.8	63.8	180–350
	2	584.6	196.4	

**Table 3:** Mean elution volumes, protein concentrations and total protein content with standard deviations of implant eluates from different surfaces and different time points.

Surface/Condition	Sample Volume (ml)	Protein Concentration (µg/mL)	Total Protein (µg)
Rough/2 min	3.5 ± 0.8	66.0 ± 30.0	231.2 ± 133.0
Rough/5 min	3.2 ± 0.3	69.2 ± 25.0	221.3 ± 93.5
Smooth/2 min	3.4 ± 0.5	13.3 ± 8.6	40.9 ± 18.6
Smooth/5 min	2.9 ± 0.6	5.1 ± 2.4	15.3 ± 8.6

**Table 4:** Shortlist of the 10 most abundant proteins eluted from the implant surfaces based on the compiled dataset (*n* = 26). These proteins were selected among the top 200 proteins identified with the highest number of peptides (based on all datasets). For each localization (intracellular/membrane, secreted to blood, ECM), proteins were ranked based on the average abundance in the respective group. Abbreviations: Avg. Abund.–ppm normalized protein abundance, ECM–extracellular matrix, #—number.

Localization	Name	# Peptides	Avg. Abund.
**Intracellular/Membrane**	Hemoglobin subunit beta	25	139,291.93
	Hemoglobin subunit alpha	25	42,991.18
	Protein AHNAK2	10	5187.38
	Keratin, type II cytoskeletal 1	21	4755.28
	Hemoglobin subunit delta	12	4421.55
	Actin, cytoplasmic 1	16	3895.84
	Carbonic anhydrase 1	19	3776.61
	Spectrin beta chain, erythrocytic	40	2911.55
	Myosin-7	99	2564.4
	Peroxiredoxin-2	16	2186.3
**Secreted to blood**	Serum albumin	99	70,046.62
	Alpha-1-antichymotrypsin	12	7424.58
	Fibrinogen beta chain	28	2523.37
	Alpha-1-antitrypsin	27	2361.18
	Fibrinogen gamma chain	26	2286.78
	Serotransferrin	49	2063.98
	Alpha-2-macroglobulin	56	1660.91
	Apolipoprotein B-100	98	1433.53
	Fibrinogen alpha chain	29	1398.07
	Complement C3	85	1241.86
**ECM**	Collagen alpha-1(II) chain	67	6790.18
	Collagen alpha-1(XXIV) chain	32	5961.83
	Collagen alpha-2(I) chain	63	3161.86
	Collagen alpha-1(XXII) chain	44	3020.04
	Collagen alpha-6(IV) chain	24	2429.28
	Collagen alpha-1(VII) chain	56	1996.28
	Collagen alpha-2(XI) chain	52	1264.12
	Collagen alpha-1(III) chain	73	1181.17
	Collagen alpha-1(V) chain	34	1154.63
	Collagen alpha-3(VI) chain	72	869.6

**Table 5:** List of the top 25 significantly enriched pathways derived from analysis of the implant proteome. Based on their location in the pathway hierarchy, pathways belonging to the same “arental pathway” were grouped, and those being highest in the hierarchy were highlighted in bold. Proteins associated with these pathways are presented in Supplementary Table S2. Abbreviation: FDR–false discovery rate.

Parental Pathway	Enriched Pathway	Total Number of Proteins in the Pathway	No. of Assigned Proteins	FDR
Extracellular matrix organization	**Extracellular matrix organization**	**418**	**54**	**7.72 × 10^−47^**
	Collagen chain trimerization	44	40	6.68 × 10^−78^
	Collagen biosynthesis and modifying enzymes	67	41	3.81 × 10^−67^
	Collagen formation	90	43	3.34 × 10^−64^
	Assembly of collagen fibrils and other multimeric structures	61	33	3.09 × 10^−51^
	Anchoring fibril formation	15	9	2.18 × 10^−14^
	Collagen degradation	89	34	2.73 × 10^−46^
	Degradation of the extracellular matrix	188	39	1.43 × 10^−41^
	Integrin cell surface interactions	109	33	5.15 × 10^−41^
	ECM proteoglycans	90	24	5.12 × 10^−28^
	Non-integrin membrane-ECM interactions	73	18	2.42 × 10^−20^
Hemostasis	**Hemostasis**	**738**	**39**	**6.04 × 10^−19^**
	Platelet degranulation	128	25	1.35 × 10^−25^
	Response to elevated platelet cytosolic Ca2+	133	25	3.44 × 10^−25^
	Platelet activation signaling and aggregation	295	27	7.54 × 10^−19^
Developmental Biology	**NCAM signaling for neurite out-growth**	**74**	**20**	**1.59 × 10^−23^**
	NCAM1 interactions	43	18	3.84 × 10^−25^
Metabolism of proteins	**Regulation of Insulin-like Growth Factor IGF transport and uptake by Insulin-like Growth Factor Binding Proteins IGFBPs**	**124**	**17**	**1.15 × 10^−14^**
	**Post-translational protein phosphorylation**	**107**	**16**	**1.90 × 10^−14^**
Signal Transduction	**Signaling by Receptor Tyrosine Kinases**	**555**	**29**	**7.95 × 10^−14^**
	Signaling by PDGF	58	20	7.84 × 10^−26^
	MET activates PTK2 signaling	30	11	1.50 × 10^−14^
	MET promotes cell motility	41	11	6.61 × 10^−13^
Immune System	**Immune System**	**2610**	**60**	**1.97 × 10^−12^**
	Neutrophil degranulation	495	26	1.97 × 10^−12^

**Table 6:** Selected proteins with significantly different abundance between 2 and 5 min exposure in situ. Differentially abundant proteins among the top 200 proteins identified with the highest number of peptides (based on all datasets) are presented. For these proteins, the respective results are also provided for the comparison between the different implant surfaces. Significant changes (*p* < 0.05) in the respective comparisons are highlighted in bold. The fold change was calculated by dividing the average abundance of the respective proteins from the case versus the control group. Abbreviations: #—number.

Name	# Peptides	Fold Change			
		Rough Surface: 5 Min vs. 2 Min	Smooth Surface: 5 Min vs. 2 Min	2 Min Exposure: Rough vs. Smooth	5 Min Exposure: Rough vs. Smooth
**Intracellular, Membrane**					
Probable E3 ubiquitin-protein ligase HECTD4	8	2.56	**8.76**	4.22	1.24
Aldehyde dehydrogenase, mitochondrial	10	0.37	**8.33**	**26.44**	1.16
Glutathione S-transferase omega-1	8	0.39	**6.53**	2.18	**0.13**
Glycerol-3-phosphate dehydrogenase [NAD(+)], cytoplasmic	9	0.89	**5.05**	**9.33**	1.65
Heat shock protein HSP 90-beta	10	0.27	**2.57**	4.49	0.48
Hemoglobin subunit gamma-2	10	1.58	**2.22**	2.35	1.67
Ankyrin-1	33	2.08	**2.17**	1.19	1.15
Hemoglobin subunit beta	25	0.93	**0.54**	0.95	**1.65**
L-lactate dehydrogenase B chain	12	0.99	**0.34**	1.36	**3.91**
Myeloperoxidase	19	0.40	**0.29**	**2.03**	**2.83**
Rab GDP dissociation inhibitor beta	8	**0.07**	0.06	**4.07**	4.63
**Secreted to blood**					
Complement C5	11	1.36	**5.60**	**9.18**	2.23
Plasminogen	15	1.34	**4.83**	**4.17**	1.15
Coagulation factor XIII A chain	10	1.06	**3.56**	0.76	**0.23**
Histidine-rich glycoprotein	9	0.80	**3.32**	0.67	**0.16**
Antithrombin-III	15	0.94	**2.16**	1.48	**0.65**
Leukocyte elastase inhibitor	10	0.71	**2.42**	**2.52**	0.74
**ECM**					
Mucin-19	10	1.86	**9.41**	11.43	2.26
Filaggrin	8	1.81	**7.15**	**8.80**	2.22
Collagen alpha-1(III) chain	73	0.45	**6.01**	**6.89**	0.52
Collagen alpha-1(XXI) chain	11	1.91	**3.41**	1.37	0.77
Collagen alpha-1(XVIII) chain	17	2.40	**3.30**	1.10	0.80
Collagen alpha-2(IX) chain	15	3.80	**3.26**	1.56	1.82
Collagen alpha-1(XXII) chain	44	0.97	**2.87**	**2.92**	0.99
Collagen alpha-3(V) chain	35	1.70	**2.32**	1.88	1.37
Collagen alpha-1(XI) chain	41	1.20	**2.27**	0.99	**0.52**
Collagen alpha-6(VI) chain	20	0.80	**0.69**	1.18	1.37
Collagen alpha-4(IV) chain	45	**1.95**	1.42	0.97	1.33

**Table 7:** List of the seven significantly enriched pathways derived from the analysis of differentially abundant proteins between 5 and 2 min exposure in situ on the smooth surface. Based on the location of the pathway in the pathway hierarchy, pathways belonging to the same “Parental pathway” were grouped, and those being highest in the hierarchy were highlighted in bold. Proteins associated with these pathways are presented in Supplementary Table S2. Abbreviation: FDR–false discovery rate.

Parental Pathway	Enriched Pathway	Total Number of Proteins in the Pathway	No. of Assigned Proteins	FDR
Extracellular matrix organization	**Collagen formation**	**90**	**9**	**1.14 × 10^−3^**
	Collagen chain trimerization	44	8	1.11 × 10^−4^
	Collagen biosynthesis and modifying enzymes	67	9	1.37 × 10^−4^
	Assembly of collagen fibrils and other multimeric structures	61	6	2.94 × 10^−2^
Hemostasis	**Common pathway of fibrin clot formation**	**22**	**4**	**2.94 × 10^−2^**
Developmental Biology	**NCAM signaling for neurite out-growth**	**74**	**7**	**1.47 × 10^−2^**
	NCAM1 interactions	43	5	3.56 × 10^−2^

**Table 8:** Selected proteins with significantly changed abundance between rough and smooth surfaces at two different exposure times in situ. Differentially abundant proteins among the top 200 proteins identified with the highest number of peptides (based on all datasets) are presented. For these proteins, the respective results are also given for the comparison between the different exposure times. Significant changes (*p* < 0.05) in the respective comparisons are highlighted in bold. The fold change was calculated by dividing the average abundance of the respective proteins from the case versus the control group. Abbreviations: #—number.

Name	# Peptides	Fold Change			
		2 Min Exposure: Rough versus Smooth	5 Min Exposure: Rough versus Smooth	Rough Surface: 5 Min vs. 2 Min	Smooth Surface: 5 Min vs. 2 Min
**Intracellular, Membrane**					
Nuclear receptor corepressor 2	8	**3.31**	2.40	0.99	1.36
14-3-3 protein epsilon	10	**2.99**	3.45	1.04	0.90
Endoplasmic reticulum chaperone BiP	10	**4.97**	1.42	0.34	1.18
Transitional endoplasmic reticulum ATPase	13	**3.87**	1.82	0.93	1.96
Eosinophil peroxidase	9	**8.63**	1.85	0.41	1.91
Four and a half LIM domains protein 1	8	**5.03**	4.08	0.63	0.78
Rab GDP dissociation inhibitor beta	8	**4.07**	4.63	0.07	0.06
Filamin-A	33	**2.97**	1.28	1.32	3.05
Protein piccolo	8	**20.26**	1.51	0.36	4.85
Glycerol-3-phosphate dehydrogenase [NAD(+)], cytoplasmic	9	**9.33**	1.65	0.89	**5.05**
Alcohol dehydrogenase 1B	8	**8.93**	6.96	1.37	1.76
Aldehyde dehydrogenase, mitochondrial	10	**26.44**	1.16	0.37	**8.33**
Protein bassoon	9	**2.82**	1.44	0.58	1.14
Tropomyosin beta chain	17	**3.20**	**6.02**	0.37	0.20
Myeloperoxidase	19	**2.03**	**2.83**	0.40	**0.29**
Malate dehydrogenase, cytoplasmic	8	**6.28**	**6.93**	0.67	0.60
Hemoglobin subunit delta	12	**2.84**	**2.58**	0.86	0.95
Histone H4	10	**2.43**	**2.52**	1.38	1.33
Keratin, type II cytoskeletal 2 epidermal	25	**0.12**	**0.09**	0.80	1.00
Myosin-7	99	**3.40**	**4.10**	0.92	0.76
Tubulin alpha-1B chain	9	**0.61**	**0.38**	0.87	1.39
Isocitrate dehydrogenase [NADP], mitochondrial	10	14.95	**Only Rough**	0.44	Only Rough
ADP/ATP translocase 1	8	5.88	**22.48**	0.72	0.19
Protein 4.1	10	13.95	**15.43**	1.98	1.79
Prelamin-A/C	17	3.98	**13.29**	1.62	0.49
ATP synthase subunit alpha, mitochondrial	12	2.52	**10.00**	0.50	0.13
Alpha-crystallin B chain	8	21.87	**7.80**	1.33	3.73
Myosin light chain 1/3, skeletal muscle isoform	9	1.92	**7.74**	2.07	0.51
Myosin light chain 3	9	2.13	**6.33**	0.52	0.17
Coronin-1A	8	10.30	**6.11**	0.27	0.46
Triosephosphate isomerase	12	6.40	**6.06**	0.84	0.89
L-lactate dehydrogenase A chain	9	3.04	**4.56**	1.51	1.01
L-lactate dehydrogenase B chain	12	1.36	**3.91**	0.99	**0.34**
Glyceraldehyde-3-phosphate dehydrogenase	12	0.95	**3.79**	0.89	0.22
Carbonic anhydrase 2	16	1.38	**3.75**	0.60	0.22
Transketolase	14	3.14	**3.35**	1.42	1.32
Vinculin	8	3.24	**3.22**	1.27	1.28
Erythrocyte membrane protein band 4.2	10	2.05	**3.00**	0.52	0.36
Pyruvate kinase PKM	10	2.06	**2.85**	1.57	1.13
Alpha-enolase	14	1.05	**2.75**	0.60	0.23
Vimentin	24	0.81	**2.57**	0.21	0.07
Fructose-bisphosphate aldolase A	13	2.17	**2.50**	0.99	0.86
Hemoglobin subunit alpha	25	1.11	**2.46**	0.87	0.40
Phosphoglycerate kinase 1	9	1.97	**2.36**	1.40	1.16
Peroxiredoxin-2	16	1.95	**2.35**	1.03	0.85
Plectin	11	0.05	**2.11**	2.05	0.05
Actin, cytoplasmic 1	16	1.52	**2.05**	0.97	0.72
Carbonic anhydrase 1	19	1.78	**2.02**	0.71	0.62
Catalase	25	1.66	**1.98**	0.98	0.82
Hemoglobin subunit beta	25	0.95	**1.65**	0.93	**0.54**
Alpha-actinin-2	31	1.06	**1.52**	0.98	0.68
Keratin, type I cytoskeletal 9	14	0.09	**0.41**	1.91	0.41
Glutathione S-transferase omega-1	8	2.18	**0.13**	0.39	**6.53**
**Secreted to blood**					
Fibronectin	34	**3.60**	1.25	0.72	2.07
Annexin A2	15	**3.15**	7.27	1.63	0.71
Leukocyte elastase inhibitor	10	**2.52**	0.74	0.71	**2.42**
Plasminogen	15	**4.17**	1.15	1.34	**4.83**
Complement C5	11	**9.18**	2.23	1.36	**5.60**
Afamin	11	**8.08**	1.77	0.78	3.55
Alpha-2-macroglobulin	56	**2.21**	1.95	1.23	1.40
Complement C3	85	**2.59**	2.39	1.19	1.28
Complement factor B	17	**3.61**	**4.34**	0.68	0.57
Alpha-1-antitrypsin	27	**2.49**	**3.18**	1.27	1.00
Apolipoprotein A-I	26	**2.54**	**1.98**	0.99	1.28
Angiotensinogen	9	**2.73**	**1.81**	1.38	2.08
Complement C4-B	43	**4.36**	**4.06**	1.02	1.10
Antithrombin-III	15	1.48	**0.65**	0.94	**2.16**
Inter-alpha-trypsin inhibitor heavy chain H2	14	4.05	**4.40**	1.04	0.96
Plasma kallikrein	12	4.04	**14.63**	2.52	0.70
Neutrophil elastase	8	3.25	**3.89**	0.98	0.82
Haptoglobin	24	1.95	**2.33**	1.46	1.21
Histidine-rich glycoprotein	9	0.67	**0.16**	0.80	**3.32**
Inter-alpha-trypsin inhibitor heavy chain H1	9	2.55	**2.55**	0.54	0.54
Hemopexin	17	0.74	**1.57**	1.13	0.54
Coagulation factor XIII A chain	10	0.76	**0.23**	1.06	**3.56**
Apolipoprotein B-100	98	1.01	**1.39**	0.65	0.47
Fibrinogen gamma chain	26	0.73	**0.49**	0.86	1.28
**ECM**					
Collagen alpha-1(II) chain	67	**1.76**	1.34	1.21	1.59
Collagen alpha-1(III) chain	73	**6.89**	0.52	0.45	**6.01**
Collagen alpha-3(IV) chain	13	**7.07**	0.27	0.45	11.56
Collagen alpha-1(XXVIII) chain	17	**2.75**	1.99	0.81	1.12
Filaggrin	8	**8.80**	2.22	1.81	**7.15**
Collagen alpha-6(IV) chain	24	**0.66**	0.51	0.90	1.18
Collagen alpha-1(XXII) chain	44	**2.92**	0.99	0.97	**2.87**
Collagen alpha-1(XV) chain	9	**3.71**	**2.62**	0.72	1.03
Decorin	9	**10.76**	**10.38**	1.39	1.44
Collagen alpha-1(XIII) chain	27	**2.26**	**2.04**	0.75	0.83
Collagen alpha-1(XIV) chain	20	1.94	**2.60**	2.00	1.50
Lumican	8	1.47	**13.21**	2.18	0.24
Collagen alpha-1(XI) chain	41	0.99	**0.52**	1.20	**2.27**

**Table 9:** List of the top 25 significantly enriched pathways derived from the analysis of differentially abundant proteins between smooth and rough surfaces. The analysis covers pathways enriched after 2 min and 5 min exposure in situ. For each comparison, the top 25 most significant pathways (based on FDR values) were shortlisted. The table presents a compilation of the shortlisted pathways, with FDR values for the pathways belonging to the top 25 highlighted in bold. Based on the location of the pathway in the pathway hierarchy, pathways belonging to the same “Parental pathway” were grouped, and those being the highest in the hierarchy were highlighted in bold. Proteins associated with these pathways are presented in Supplementary Table S2. Abbreviation: FDR–false discovery rate.

**Parental Pathway**	**Enriched Pathway**	**Total Number of Proteins in the Pathway**	**After 2 Min Exposure**		**After 5 Min Exposure**	
			FDR	No. of Assigned Proteins	FDR	No. of Assigned Proteins
Extracellular matrix organization	**Extracellular matrix organization**	**418**	**1.96 × 10^−5^**	**17**	**2.20 × 10^−3^**	**18**
	Collagen chain trimerization	44	**2.40 × 10^−6^**	8	3.34 × 10^−2^	4
	Collagen biosynthesis and modifying enzymes	67	**1.87 × 10^−5^**	8	-	-
	Collagen formation	90	**1.87 × 10^−5^**	9	-	-
	Degradation of the extracellular matrix	188	**1.87 × 10^−5^**	12	3.78 × 10^−3^	11
	Non-integrin membrane-ECM interactions	73	**2.37 × 10^−4^**	7	-	-
	Collagen degradation	89	**7.86 × 10^−4^**	7	2.15 × 10^−2^	6
	ECM proteoglycans	90	**7.86 × 10^−4^**	7	-	-
	Assembly of collagen fibrils and other multimeric structures	61	**7.89 × 10^−4^**	6	-	-
	Integrin cell surface interactions	109	**9.73 × 10^−3^**	6	-	-
Hemostasis	**Hemostasis**	**738**	**7.10 × 10^−3^**	**17**	**3.16 × 10^−6^**	**33**
	Platelet degranulation	128	**1.65 × 10^−4^**	9	**1.16 × 10^−6^**	14
	Response to elevated platelet cytosolic Ca^2+^	133	**1.99 × 10^−4^**	9	**1.72 × 10^−6^**	14
	Platelet activation signaling and aggregation	295	**7.10 × 10^−3^**	10	**1.50 × 10^−4^**	17
Metabolism of proteins	**Regulation of Insulin-like Growth Factor IGF transport and uptake by Insulin-like Growth Factor Binding Proteins IGFBPs**	**124**	**4.27 × 10^−3^**	**7**	**7.15 × 10^−4^**	**10**
Signal Transduction	**MET activates PTK2 signaling**	**30**	**4.29 × 10^−3^**	**4**	-	-
	**MAPK family signaling cascades**	**299**	-	-	**1.72 × 10^−4^**	**17**
	RAF/MAP kinase cascade	248	-	-	1.88 × 10^−5^	17
	MAPK1/MAPK3 signaling	254	-	-	2.49 × 10^−5^	17
Immune System	**Immune System**	**2610**	**4.27 × 10^−3^**	**41**	**1.22 × 10^−5^**	**73**
	Neutrophil degranulation	495	**3.63 × 10^−5^**	18	**9.16 × 10^−11^**	34
	Gene and protein expression by JAK-STAT signaling after Interleukin-12 stimulation	39	**1.05 × 10^−3^**	5	2.41 × 10^−2^	4
	Interleukin-12 signaling	56	**4.29 × 10^−3^**	5	-	-
	Activation of C3 and C5	12	**4.33 × 10^−3^**	3	-	-
	Innate Immune System	1313	2.15 × 10^−2^	23	**4.18 × 10^−8^**	52
	Signaling by Interleukins	706	4.41 × 10^−2^	14	**3.32 × 10^−6^**	32
	Cytokine Signaling in Immune system	983	-	-	**6.30 × 10^−5^**	36
	FLT3 Signaling	275	-	-	**6.37 × 10^−5^**	17
	Other interleukin signaling	298	-	-	**3.16 × 10^−6^**	20
Muscle contraction	**Muscle contraction**	**216**	4.29 × 10^−3^	**9**	**3.16 × 10^−6^**	**17**
	Smooth Muscle Contraction	42	1.41 × 10^−3^	5	7.50 × 10^−3^	5
	Striated Muscle Contraction	36	7.10 × 10^−3^	4	**6.01 × 10^−11^**	12
Transport of small molecules	**Erythrocytes take up oxygen and release carbon dioxide**	**9**	-	-	**2.71 × 10^−4^**	**4**
Vesicle-mediated transport	**Binding and Uptake of Ligands by Scavenger Receptors**	**112**	**4.15 × 10^−2^**	**5**	**3.50 × 10^−4^**	**10**
Metabolism	**Metabolism**	**2262**	-	-	**6.39 × 10^−8^**	**73**
	Metabolism of carbohydrates	312	-	-	**8.30 × 10^−12^**	29
	Glucose metabolism	93	-	-	**2.81 × 10^−9^**	15
	Gluconeogenesis	34	4.26 × 10^−2^	3	**1.16 × 10^−8^**	10
	Glycolysis	73	-	-	**1.33 × 10^−7^**	12
Programmed Cell Death	**Programmed Cell Death**	**193**	-	-	**8.29 × 10^−5^**	**14**
	Apoptosis	186	-	-	**6.23 × 10^−5^**	14
	Activation of BH3-only proteins	32	**5.02 × 10^−3^**	4	-	-

## Data Availability

The processed data required to reproduce these findings are available to download from the supporting information.

## Supplementary Materials

The following supporting information can be downloaded at: https://www.mdpi.com/article/10.3390/jfb13020044/s1, Supplementary Table S1: List of proteins identified with at least two peptides, Supplementary Table S2: List of significantly enriched pathways, Supplementary File A: Full version of the tables: Tables in this article have been decreased in complexity. Complete versions of these tables can be found here.
