# Supplementary material for: The Implant Proteome—The Right Surgical Glue to Fix Titanium Implants In Situ
Source: J Funct Biomater. 2022 Apr 15;13(2):44. doi: 10.3390/jfb13020044 (PMC9036294; doi:10.3390/jfb13020044)
Supplement: Supplementary file 1 [file jfb-13-00044-s001.zip › jfb-1658391-Supplementary File A.pdf]

Full version of the tables

Tables in this article have been decreased in complexity. Complete versions of these tables can be found here:

Table 4-S. Shortlist of the 10 most abundant proteins eluted from the implant surfaces after different incubation times. These proteins were selected among top 200 proteins identified with the highest number of peptides (based on all datasets). For each localization (intracellular/membrane, secreted to blood, ECM), proteins were ranked based on the average abundance in the respective group. The rank of proteins belonging to the top 10 with the highest abundance is in bold. Proteins are sorted based on the abundance rank in the compiled dataset. Abbreviations: Avg. Abund. – ppm normalised protein abundance, ECM – extracellular matrix.

Name	# peptides	Compiled dataset (n=26)		Rough surface, 2min (n=7)		Rough surface, 5min (n=6)		Smooth surface, 2min (n=7)		Smooth surface, 5min (n=6)	
		Avg. Abund.	Rank	Avg. Abund.	Rank	Avg. Abund.	Rank	Avg. Abund.	Rank	Avg. Abund.	Rank
**Intracellular/Membrane**											
Hemoglobin subunit beta	25	139291.93	**1**	154876.33	**1**	144721.00	**1**	163403.4	**1**	87550.99	**1**
Hemoglobin subunit alpha	25	42991.18	**2**	54101.03	**2**	47291.11	**2**	48580.9	**2**	19208.4	**2**
Protein AHNAK2	10	5187.38	**3**	8124.87	**3**	2044.41	**10**	4790.61	**5**	5366.17	**4**
Keratin, type II cytoskeletal 1	21	4755.28	**4**	4662.82	**7**	2663.98	**9**	5921.46	**3**	5593.91	**3**
Hemoglobin subunit delta	12	4421.55	**5**	6909.44	**4**	5956.87	**3**	2431.57	**8**	2305.33	**6**
Actin, cytoplasmic 1	16	3895.84	**6**	4989.7	**6**	4864.7	**4**	3279.31	**6**	2370.12	**5**
Carbonic anhydrase 1	19	3776.61	**7**	5678.07	**5**	4021.49	**5**	3192.65	**7**	1994.63	**7**
Spectrin beta chain, erythrocytic	40	2911.55	**8**	1171.39	15	3219.65	**7**	5547.45	**4**	1558.4	**8**
Myosin-7	99	2564.4	**9**	4190.33	**8**	3847.96	**6**	1232.44	11	937.86	12
Peroxiredoxin-2	16	2186.3	**10**	2928.72	**9**	3019.12	**8**	1504.49	**9**	1282.77	**9**
Carbonic anhydrase 2	16	989.25	11	1546.26	**10**	929.53	16	1118.97	12	247.78	29
Titin	75	891.83	15	721.44	20	1116.26	13	655.53	20	1141.86	**10**
Vimentin	24	797.95	16	1191.67	13	253.5	38	1470.35	**10**	98.57	44
**Secreted to blood**											
Serum albumin	99	70046.62	**1**	79197.19	**1**	71030.02	**1**	66819.75	**1**	62152.23	**1**
Alpha-1-antichymotrypsin	12	7424.58	**2**	4625.31	**2**	4935.14	**2**	11778.05	**2**	8100.8	**2**
Fibrinogen beta chain	28	2523.37	**3**	2746.04	**5**	1780.6	**7**	2304.23	**4**	3262.02	**3**
Alpha-1-antitrypsin	27	2361.18	**4**	3092.83	**4**	3936	**3**	1241.14	**8**	1239.48	**8**
Fibrinogen gamma chain	26	2286.78	**5**	1842.04	**7**	1588.86	**9**	2526.6	**3**	3223.76	**4**
Serotransferrin	49	2063.98	**6**	3117.23	**3**	2066.26	**5**	1488.17	**7**	1504.69	**6**
Alpha-2-macroglobulin	56	1660.91	**7**	2021.85	**6**	2494.49	**4**	914.1	**9**	1277.52	**7**
Apolipoprotein B-100	98	1433.53	**8**	1805.88	**8**	1172.19	11	1788.95	**5**	845.83	**10**

Fibrinogen alpha chain	29	1398.07	**9**	1190.19	11	1046.5	12	1716.15	**6**	1621.09	**5**
Complement C3	85	1241.86	**10**	1630.82	**10**	1934.62	**6**	629.95	11	809.19	11
Apolipoprotein A-I	26	1215.53	11	1686.24	**9**	1677.63	**8**	663.69	**10**	848.07	**9**
Haptoglobin	24	844.55	12	951.8	12	1385.04	**10**	489.07	13	593.65	13
**ECM**											
Collagen alpha-1(II) chain	67	6790.18	**1**	7460.49	**1**	9029.08	**1**	4242.75	**2**	6741.27	**1**
Collagen alpha-1(XXIV) chain	32	5961.83	**2**	6165.53	**2**	6751.05	**2**	6632.97	**1**	4151.97	**2**
Collagen alpha-2(I) chain	63	3161.86	**3**	4888.26	**3**	3259.09	**4**	2581.8	**4**	1727.23	**7**
Collagen alpha-1(XXII) chain	44	3020.04	**4**	3711.8	**4**	3618.18	**3**	1273.31	**7**	3652.71	**3**
Collagen alpha-6(IV) chain	24	2429.28	**5**	1880	**6**	1688.34	**6**	2833.78	**3**	3339.13	**4**
Collagen alpha-1(VII) chain	56	1996.28	**6**	1277.07	**7**	1866.92	**5**	1819.9	**5**	3170.47	**5**
Collagen alpha-2(XI) chain	52	1264.12	**7**	593.05	12	1083.86	**9**	945.27	**8**	2599.27	**6**
Collagen alpha-1(III) chain	73	1181.17	**8**	1924.21	**5**	866.5	11	279.47	19	1680.94	**8**
Collagen alpha-1(V) chain	34	1154.63	**9**	735.27	**9**	1084.91	**8**	1275.41	**6**	1572.68	**9**
Collagen alpha-3(VI) chain	72	869.6	**10**	634.54	11	924.04	**10**	894.08	**9**	1060.83	11
Collagen alpha-1(XXVII) chain	31	763.05	11	685.98	**10**	1110.34	**7**	650.47	**10**	637.01	17
Collagen alpha-1(XIX) chain	31	749.88	12	1114.74	**8**	818.81	12	195.3	21	902.27	14
Collagen alpha-2(IV) chain	31	676.35	13	423.96	13	587.31	16	631.05	11	1112.69	**10**

Table 6-S. Selected proteins with significantly different abundance between 2 and 5 min exposure *in situ*. Differentially abundant proteins among top 200 proteins identified with the highest number of peptides (based on all datasets) are presented. For these proteins, respective results are also provided for the comparison between the different implant surfaces. Significant changes ($p<0.05$) in the respective comparisons are highlighted in bold. Fold change was calculated by dividing the average abundance of the respective proteins from the case versus the control group.

Name	# peptides	Rough surface: 5 min vs 2 min		Smooth surface: 5 min vs 2 min		2 min exposure: Rough vs Smooth		5 min exposure: Rough vs Smooth	
		**Fold change**	**p-value**	**Fold change**	**p-value**	**Fold change**	**p-value**	**Fold change**	**p-value**
**Intracellular, Membrane**									
Probable E3 ubiquitin-protein ligase HECTD4	8	2.56	0.10	**8.76**	**0.04**	4.22	0.20	1.24	0.84
Aldehyde dehydrogenase, mitochondrial	10	0.37	0.73	**8.33**	**0.02**	**26.44**	**0.05**	1.16	0.69
Glutathione S-transferase omega-1	8	0.39	0.56	**6.53**	**0.00**	2.18	0.55	**0.13**	**0.03**
Glycerol-3-phosphate dehydrogenase [NAD(+)], cytoplasmic	9	0.89	0.94	**5.05**	**0.03**	**9.33**	**0.03**	1.65	0.56
Heat shock protein HSP 90-beta	10	0.27	0.14	**2.57**	**0.01**	4.49	0.08	0.48	0.16
Hemoglobin subunit gamma-2	10	1.58	0.53	**2.22**	**0.03**	2.35	0.08	1.67	0.22
Ankyrin-1	33	2.08	0.10	**2.17**	**0.04**	1.19	0.94	1.15	1.00
Hemoglobin subunit beta	25	0.93	0.63	**0.54**	**0.00**	0.95	0.94	**1.65**	**0.03**
L-lactate dehydrogenase B chain	12	0.99	0.84	**0.34**	**0.03**	1.36	0.38	**3.91**	**0.03**
Myeloperoxidase	19	0.40	0.05	**0.29**	**0.00**	**2.03**	**0.02**	**2.83**	**0.03**
Rab GDP dissociation inhibitor beta	8	**0.07**	**0.01**	0.06	0.35	**4.07**	**0.03**	4.63	0.06
**Secreted to blood**									
Complement C5	11	1.36	0.52	**5.60**	**0.01**	**9.18**	**0.03**	2.23	0.22
Plasminogen	15	1.34	0.62	**4.83**	**0.02**	**4.17**	**0.03**	1.15	1.00
Coagulation factor XIII A chain	10	1.06	0.84	**3.56**	**0.01**	0.76	0.47	**0.23**	**0.03**
Histidine-rich glycoprotein	9	0.80	0.94	**3.32**	**0.04**	0.67	0.30	**0.16**	**0.03**
Antithrombin-III	15	0.94	1.00	**2.16**	**0.01**	1.48	0.08	**0.65**	**0.03**
Leukocyte elastase inhibitor	10	0.71	0.37	**2.42**	**0.01**	**2.52**	**0.03**	0.74	0.44
**ECM**									
Mucin-19	10	1.86	0.47	**9.41**	**0.02**	11.43	0.21	2.26	0.22
Filaggrin	8	1.81	0.84	**7.15**	**0.02**	**8.80**	**0.02**	2.22	0.69
Collagen alpha-1(III) chain	73	0.45	0.37	**6.01**	**0.04**	**6.89**	**0.02**	0.52	1.00
Collagen alpha-1(XXI) chain	11	1.91	0.07	**3.41**	**0.00**	1.37	0.58	0.77	0.16
Collagen alpha-1(XVIII) chain	17	2.40	0.10	**3.30**	**0.03**	1.10	0.80	0.80	0.16
Collagen alpha-2(IX) chain	15	3.80	0.05	**3.26**	**0.04**	1.56	0.67	1.82	0.31
Collagen alpha-1(XXII) chain	44	0.97	0.95	**2.87**	**0.01**	**2.92**	**0.05**	0.99	0.84
Collagen alpha-3(V) chain	35	1.70	0.14	**2.32**	**0.04**	1.88	0.11	1.37	0.31
Collagen alpha-1(XI) chain	41	1.20	0.45	**2.27**	**0.03**	0.99	0.94	**0.52**	**0.03**
Collagen alpha-6(VI) chain	20	0.80	0.84	**0.69**	**0.04**	1.18	0.30	1.37	0.16
Collagen alpha-4(IV) chain	45	**1.95**	**0.01**	1.42	0.35	0.97	0.94	1.33	0.31

Table 8-S. Selected proteins with significantly changed abundance between rough and smooth surfaces at two different exposure times *in situ*. Differentially abundant proteins among top 200 proteins identified with the highest number of peptides (based on all datasets) are presented. For these proteins, respective results are also given for the comparison between the different exposure times. Significant changes (p<0.05) in the respective comparisons are highlighted in bold. Fold change was calculated by dividing the average abundance of the respective proteins from the case versus the control group.

Name	# peptides	2 min exposure: Rough versus Smooth		5 min exposure: Rough versus Smooth		Rough surface: 5 min vs 2 min		Smooth surface: 5 min vs 2 min	
		Fold change	p-value	Fold change	p-value	Fold change	p-value	Fold change	p-value
**Intracellular, Membrane**									
Nuclear receptor corepressor 2	8	**3.31**	**0.02**	2.40	0.09	0.99	0.95	1.36	0.43
14-3-3 protein epsilon	10	**2.99**	**0.02**	3.45	0.06	1.04	0.84	0.90	0.83
Endoplasmic reticulum chaperone BiP	10	**4.97**	**0.02**	1.42	0.84	0.34	0.09	1.18	0.71
Transitional endoplasmic reticulum ATPase	13	**3.87**	**0.02**	1.82	0.09	0.93	0.73	1.96	0.31
Eosinophil peroxidase	9	**8.63**	**0.03**	1.85	0.21	0.41	0.07	1.91	0.51
Four and a half LIM domains protein 1	8	**5.03**	**0.03**	4.08	0.06	0.63	0.29	0.78	0.94
Rab GDP dissociation inhibitor beta	8	**4.07**	**0.03**	4.63	0.06	**0.07**	**0.01**	0.06	0.35
Filamin-A	33	**2.97**	**0.03**	1.28	0.31	1.32	0.37	3.05	0.62
Protein piccolo	8	**20.26**	**0.03**	1.51	0.69	0.36	0.62	4.85	0.09
Glycerol-3-phosphate dehydrogenase [NAD(+)], cytoplasmic	9	**9.33**	**0.03**	1.65	0.56	0.89	0.94	**5.05**	**0.03**
Alcohol dehydrogenase 1B	8	**8.93**	**0.03**	6.96	0.09	1.37	0.52	1.76	0.31
Aldehyde dehydrogenase, mitochondrial	10	**26.44**	**0.05**	1.16	0.69	0.37	0.73	**8.33**	**0.02**
Protein bassoon	9	**2.82**	**0.05**	1.44	0.44	0.58	0.23	1.14	0.94
Tropomyosin beta chain	17	**3.20**	**0.02**	**6.02**	**0.03**	0.37	0.45	0.20	0.83
Myeloperoxidase	19	**2.03**	**0.02**	**2.83**	**0.03**	0.40	0.05	**0.29**	**0.00**
Malate dehydrogenase, cytoplasmic	8	**6.28**	**0.03**	**6.93**	**0.03**	0.67	0.84	0.60	0.51
Hemoglobin subunit delta	12	**2.84**	**0.03**	**2.58**	**0.03**	0.86	0.73	0.95	0.63
Histone H4	10	**2.43**	**0.03**	**2.52**	**0.03**	1.38	0.37	1.33	0.63
Keratin, type II cytoskeletal 2 epidermal	25	**0.12**	**0.03**	**0.09**	**0.03**	0.80	0.73	1.00	0.73
Myosin-7	99	**3.40**	**0.03**	**4.10**	**0.03**	0.92	0.95	0.76	0.84
Tubulin alpha-1B chain	9	**0.61**	**0.02**	**0.38**	**0.03**	0.87	0.45	1.39	0.18
Isocitrate dehydrogenase [NADP], mitochondrial	10	14.95	0.10	**Only Rough**	**0.03**	0.44	0.94	Only Rough	0.21
ADP/ATP translocase 1	8	5.88	0.05	**22.48**	**0.04**	0.72	0.89	0.19	0.14
Protein 4.1	10	13.95	0.10	**15.43**	**0.03**	1.98	0.35	1.79	0.47
Prelamin-A/C	17	3.98	0.21	**13.29**	**0.03**	1.62	0.28	0.49	0.59
ATP synthase subunit alpha, mitochondrial	12	2.52	0.27	**10.00**	**0.03**	0.50	0.94	0.13	0.51
Alpha-crystallin B chain	8	21.87	0.05	**7.80**	**0.03**	1.33	0.35	3.73	0.63

Myosin light chain 1/3, skeletal muscle isoform	9	1.92	0.20	**7.74**	**0.03**	2.07	0.14	0.51	0.88
Myosin light chain 3	9	2.13	0.30	**6.33**	**0.03**	0.52	0.45	0.17	1.00
Coronin-1A	8	10.30	0.11	**6.11**	**0.03**	0.27	0.53	0.46	0.82
Triosephosphate isomerase	12	6.40	0.16	**6.06**	**0.03**	0.84	0.95	0.89	1.00
L-lactate dehydrogenase A chain	9	3.04	0.11	**4.56**	**0.03**	1.51	0.23	1.01	0.83
L-lactate dehydrogenase B chain	12	1.36	0.38	**3.91**	**0.03**	0.99	0.84	**0.34**	**0.03**
Glyceraldehyde-3-phosphate dehydrogenase	12	0.95	0.58	**3.79**	**0.03**	0.89	0.84	0.22	0.10
Carbonic anhydrase 2	16	1.38	0.30	**3.75**	**0.03**	0.60	0.10	0.22	0.14
Transketolase	14	3.14	0.16	**3.35**	**0.03**	1.42	0.37	1.32	0.66
Vinculin	8	3.24	0.11	**3.22**	**0.03**	1.27	0.45	1.28	0.61
Erythrocyte membrane protein band 4.2	10	2.05	0.11	**3.00**	**0.03**	0.52	0.14	0.36	0.23
Pyruvate kinase PKM	10	2.06	0.30	**2.85**	**0.03**	1.57	0.63	1.13	0.62
Alpha-enolase	14	1.05	0.30	**2.75**	**0.03**	0.60	0.37	0.23	0.62
Vimentin	24	0.81	0.94	**2.57**	**0.03**	0.21	0.29	0.07	0.05
Fructose-bisphosphate aldolase A	13	2.17	0.30	**2.50**	**0.03**	0.99	0.45	0.86	0.62
Hemoglobin subunit alpha	25	1.11	0.58	**2.46**	**0.03**	0.87	0.53	0.40	0.10
Phosphoglycerate kinase 1	9	1.97	0.22	**2.36**	**0.03**	1.40	0.29	1.16	0.52
Peroxiredoxin-2	16	1.95	0.16	**2.35**	**0.03**	1.03	0.84	0.85	0.63
Plectin	11	0.05	0.93	**2.11**	**0.03**	2.05	0.10	0.05	0.35
Actin, cytoplasmic 1	16	1.52	0.30	**2.05**	**0.03**	0.97	1.00	0.72	1.00
Carbonic anhydrase 1	19	1.78	0.22	**2.02**	**0.03**	0.71	0.37	0.62	0.05
Catalase	25	1.66	0.08	**1.98**	**0.03**	0.98	0.95	0.82	0.37
Hemoglobin subunit beta	25	0.95	0.94	**1.65**	**0.03**	0.93	0.63	**0.54**	**0.00**
Alpha-actinin-2	31	1.06	0.81	**1.52**	**0.03**	0.98	0.63	0.68	0.23
Keratin, type I cytoskeletal 9	14	0.09	0.08	**0.41**	**0.03**	1.91	0.62	0.41	0.63
Glutathione S-transferase omega-1	8	2.18	0.55	**0.13**	**0.03**	0.39	0.56	**6.53**	**0.00**
**Secreted to blood**									
Fibronectin	34	**3.60**	**0.02**	1.25	0.56	0.72	0.23	2.07	0.18
Annexin A2	15	**3.15**	**0.02**	7.27	0.09	1.63	1.00	0.71	0.83
Leukocyte elastase inhibitor	10	**2.52**	**0.03**	0.74	0.44	0.71	0.37	**2.42**	**0.01**
Plasminogen	15	**4.17**	**0.03**	1.15	1.00	1.34	0.62	**4.83**	**0.02**
Complement C5	11	**9.18**	**0.03**	2.23	0.22	1.36	0.52	**5.60**	**0.01**
Afamin	11	**8.08**	**0.03**	1.77	0.31	0.78	0.72	3.55	0.10
Alpha-2-macroglobulin	56	**2.21**	**0.05**	1.95	0.09	1.23	0.45	1.40	0.29
Complement C3	85	**2.59**	**0.05**	2.39	0.06	1.19	0.84	1.28	0.63
Complement factor B	17	**3.61**	**0.02**	**4.34**	**0.03**	0.68	0.29	0.57	0.72
Alpha-1-antitrypsin	27	**2.49**	**0.05**	**3.18**	**0.03**	1.27	0.37	1.00	0.37
Apolipoprotein A-I	26	**2.54**	**0.05**	**1.98**	**0.03**	0.99	0.84	1.28	0.37
Angiotensinogen	9	**2.73**	**0.03**	**1.81**	**0.03**	1.38	0.43	2.08	0.22
Complement C4-B	43	**4.36**	**0.05**	**4.06**	**0.03**	1.02	0.84	1.10	0.43
Antithrombin-III	15	1.48	0.08	**0.65**	**0.03**	0.94	1.00	**2.16**	**0.01**
Inter-alpha-trypsin inhibitor heavy chain H2	14	4.05	0.16	**4.40**	**0.03**	1.04	0.84	0.96	0.83

Plasma kallikrein	12	4.04	0.21	**14.63**	**0.03**	2.52	0.28	0.70	0.82
Neutrophil elastase	8	3.25	0.22	**3.89**	**0.03**	0.98	0.84	0.82	0.94
Haptoglobin	24	1.95	0.30	**2.33**	**0.03**	1.46	0.45	1.21	0.73
Histidine-rich glycoprotein	9	0.67	0.30	**0.16**	**0.03**	0.80	0.94	**3.32**	**0.04**
Inter-alpha-trypsin inhibitor heavy chain H1	9	2.55	0.35	**2.55**	**0.03**	0.54	0.84	0.54	0.94
Hemopexin	17	0.74	0.47	**1.57**	**0.03**	1.13	0.95	0.54	0.37
Coagulation factor XIII A chain	10	0.76	0.47	**0.23**	**0.03**	1.06	0.84	**3.56**	**0.01**
Apolipoprotein B-100	98	1.01	0.69	**1.39**	**0.03**	0.65	0.14	0.47	1.00
Fibrinogen gamma chain	26	0.73	0.22	**0.49**	**0.03**	0.86	0.45	1.28	0.45
**ECM**									
Collagen alpha-1(II) chain	67	**1.76**	**0.02**	1.34	0.44	1.21	0.95	1.59	0.45
Collagen alpha-1(III) chain	73	**6.89**	**0.02**	0.52	1.00	0.45	0.37	**6.01**	**0.04**
Collagen alpha-3(IV) chain	13	**7.07**	**0.02**	0.27	0.56	0.45	0.37	11.56	0.17
Collagen alpha-1(XXVIII) chain	17	**2.75**	**0.02**	1.99	0.44	0.81	1.00	1.12	0.35
Filaggrin	8	**8.80**	**0.02**	2.22	0.69	1.81	0.84	**7.15**	**0.02**
Collagen alpha-6(IV) chain	24	**0.66**	**0.03**	0.51	0.06	0.90	0.95	1.18	0.63
Collagen alpha-1(XXII) chain	44	**2.92**	**0.05**	0.99	0.84	0.97	0.95	**2.87**	**0.01**
Collagen alpha-1(XV) chain	9	**3.71**	**0.02**	**2.62**	**0.03**	0.72	1.00	1.03	0.22
Decorin	9	**10.76**	**0.02**	**10.38**	**0.03**	1.39	0.37	1.44	0.94
Collagen alpha-1(XIII) chain	27	**2.26**	**0.05**	**2.04**	**0.03**	0.75	0.45	0.83	0.72
Collagen alpha-1(XIV) chain	20	1.94	0.21	**2.60**	**0.03**	2.00	0.10	1.50	0.22
Lumican	8	1.47	0.26	**13.21**	**0.03**	2.18	0.10	0.24	0.61
Collagen alpha-1(XI) chain	41	0.99	0.94	**0.52**	**0.03**	1.20	0.45	**2.27**	**0.03**
